# Gunshot Wound of the Heart, without Perforation of the Pericarduim

**Published:** 1846-04

**Authors:** 


*Gunshot wound of the heart, without perforation of the Pericarduim.*—Professor Holmes records, in the *British American Journal,* a case which seems to show the possibility of such an occurrence. An individual had received a gun-shot wound of the left side of the chest, and died soon after. On removing the anterior walls of this cavity, "the appearances presented were, a bloody ecchymosed condition of the anterior part of the left lung, as it laps over the pericardium, a bloody and infiltrated state of the cellular substances lying on the pericardium, and an ecchymosis of the extent of about an inch and a half, filling the anterior edge of the right lung, where it lies in contact with the pericardium. The pericardium evidently contained a large quantity of fluid, the nature of which was denoted by the colour of the membrane."

The lead which had caused the injury was found in the right pleural cavity, but its course could not be traced. No perforation of the pericardium, which was filled with blood and serum, could be found; but on its being laid open,

"There was seen on the anterior wall of the heart, penetrating the right ventricle, a transverse linear opening without laceration at the margins, which were smooth, and rather turned inwards, and sufficiently large to admit the finger."

The difficulty of explaining this occurrence is considered by the writer; and he first argues that this injury was not likely to be the result of a spontaneous rupture. He refers to cases recorded by writers on military surgery, which show that pieces of linen and other clothes have been sometimes driven without being torn, into wounds; and thus that balls have been withdrawn inclosed in a kind of purse. Somewhat similar is the author's explanation of the present occurrence:—

"Entertaining," he says, "no doubt that the wound was caused by the direct contact of the ball, driving the pericardium before it, I think the manner of its formation may be more readily understood by supposing that at the instant of being struck, the heart was in the act of contraction, its fibres hard and rigid from their muscular action. In this state the ball suddenly impinging produced an effect similar to what happens to an over-braced harp-string when struck. The fibres snapped across."—*London Lancet.*

